# A novel shape-based approach to identify gestational age-adjusted growth patterns from birth to 11 years of age

**DOI:** 10.1038/s41598-023-28485-4

**Published:** 2023-01-31

**Authors:** Lorena López-Domínguez, Diego G. Bassani, Celine Bourdon, Paraskevi Massara, Iná S. Santos, Alicia Matijasevich, Aluísio. J. D. Barros, Elena M. Comelli, Robert H. J. Bandsma

**Affiliations:** 1 Department of Nutritional Sciences, Temerty Faculty of Medicine, University of Toronto, 1 King’s College Circle, Medical Sciences Building, Toronto, ON M5S 1A8 Canada; 2 Translational Medicine Program, Hospital for Sick Children, Peter Gilgan Centre for Research and Learning, 686 Bay Street, Toronto, ON M5G 0A4 Canada; 3 Department of Epidemiology, Dalla Lana School of Public Health, University of Toronto, Toronto, ON Canada; 4 Centre for Global Child Health, Child Health Evaluative Sciences, Hospital for Sick Children, Toronto, ON Canada; 5 Division of Paediatric Medicine, Hospital for Sick Children, Toronto, ON Canada; 6 The Childhood Acute Illness & Nutrition Network, Nairobi, Kenya; 7 Post-Graduate Program in Epidemiology, Federal University of Pelotas, Pelotas, RS Brazil; 8 Departamento de Medicina Preventiva, Faculdade de Medicina FMUSP, Universidade de São Paulo, São Paulo, Brazil; 9 Joannah and Brian Lawson Center for Child Nutrition, University of Toronto, Toronto, ON Canada; 10 Division of Gastroenterology, Hepatology and Nutrition, Hospital for Sick Children, Toronto, ON Canada

**Keywords:** Translational research, Epidemiology

## Abstract

Child growth patterns assessment is critical to design public health interventions. However, current analytical approaches may overlook population heterogeneity. To overcome this limitation, we developed a growth trajectories clustering pipeline that incorporates a shape-respecting distance, baseline centering (i.e., birth-size normalized trajectories) and Gestational Age (GA)-correction to characterize shape-based child growth patterns. We used data from 3945 children (461 preterm) in the 2004 Pelotas Birth Cohort with at least 3 measurements between birth (included) and 11 years of age. Sex-adjusted weight-, length/height- and body mass index-for-age z-scores were derived at birth, 3 months, and at 1, 2, 4, 6 and 11 years of age (INTERGROWTH-21st and WHO growth standards). Growth trajectories clustering was conducted for each anthropometric index using k-means and a shape-respecting distance, accounting or not for birth size and/or GA-correction. We identified 3 trajectory patterns for each anthropometric index: increasing (*High*), stable (*Middle*) and decreasing (*Low*). Baseline centering resulted in pattern classification that considered early life growth traits. GA-correction increased the intercepts of preterm-born children trajectories, impacting their pattern classification. Incorporating shape-based clustering, baseline centering and GA-correction in growth patterns analysis improves the identification of subgroups meaningful for public health interventions.

## Introduction

Childhood growth is a routinely tracked marker of optimal health and development^1^. Cohort studies provide longitudinal measurements at various time points that are typically used to represent growth in a population using trajectories. Traditionally, individual child trajectories are summarized into a single average trajectory for the population under study. Although an average growth trajectory can be representative of the population, reducing the entire population to a single average trajectory can disregard details of growth patterns related to the heterogeneity in growth^2^.

To address this limitation, clustering can be used to uncover the inherent heterogeneity of growth data. This group-based approach relies on algorithms to classify individuals and allows to identify meaningful subgroups of individuals who share common characteristics over time^3^. There is no consensus on the best method to use for clustering trajectories^4^, k-means is commonly employed because of simplicity and flexibility. Variations of k-means use different distance metrics, which evaluate the difference between each pair of elements. Most classical distances (e.g., Euclidean, Manhattan) assess the similarity of values at each time point of a trajectory. However, growth trajectories are non-linear and the intervals available for each individual in a cohort study are often variable. Distances that consider the whole shape of the trajectory can help capture the progression of growth over time, accounting for heterogeneity and summarizing subgroups of growth trajectories in a population. Growth trajectory clusters can also potentially be influenced by the intercept (i.e., start point of the trajectory), which can mask part of the heterogeneity in the population by grouping together individuals with a closer intercept but that do not share a similar shape.

The World Health Organization (WHO) Child Growth Standards^5,6^ are the most widely applied international standards for evaluating postnatal growth in children worldwide; however, they are based on a reference population of term-born children^7^. The International Fetal and Newborn Growth Consortium for the 21st Century (INTERGROWTH-21st) newborn size standards have been developed to account for gestational age (GA) in the application of growth standards until 64 weeks' postmenstrual age^8^. Applying GA-correction to growth standards improves the understanding of growth and the associated outcomes, especially in Low- and Middle-Income Countries^9^. However, the impact of GA-correction in the context of clustering childhood growth trajectories has not been investigated.

We aimed to implement a pipeline for clustering growth trajectories that incorporates a shape-respecting distance, baseline centering (i.e., normalizing the trajectory intercept to account for birth size) in combination with GA-correction to identify shape-based child growth patterns. We anticipated that this approach would allow for a characterization of childhood growth patterns that capture the full spectrum of heterogeneity in growth trajectories.

## Results

### Participant’s characteristics

We studied 3945 children from the 2004 Pelotas birth cohort in which children were recruited at birth and followed up longitudinally at months 3, 12, 24, and 48, and at 6 and 11 years of age. Table 1 shows the characteristics of the 3945 all participants and those born preterm only (n = 461). The mean birth weight was 3.2 (± 0.5) kg and the mean birth length was 48.3 (± 2.4) cm. Mean gestational age of term-born children was 39.4 (± 1.99) weeks and 8.3% (n = 329) of children classified as Low Birth Weight (< 2500 g) (n = 12 Very Low Birth Weight (< 1500 g)). The sample comprised 1903 (48.2%) females. The mean difference between GA-corrected-age and uncorrected age was of 1.08 months.

**Table 1:** Characteristics of participants from the 2004 Pelotas Birth cohort included in this analysis, 2004–2015.

	Total n = 3945	Preterm birth, < 37 weeks GA n = 416 (11.7%)
Female sex, n (%)	1903 (48%)	234 (12%)
Gestational age, weeks, mean ± SD	38.9 ± 2.4	35.2 ± 2.0
Birth weight, g, mean ± SD	318.5 ± 50.8	258.1 ± 52.7
Birth length, cm, mean ± SD	48.3 ± 2.4	45.6 ± 2.9
Low birth weight (< 2500 g), n (%)	329 (8.3%)	183 (39.7%)
GA-corrected age, months, mean ± SD		
Follow up 1		3.0 ± 0.1
Follow up 2		11.9 ± 0.2
Follow up 3		23.9 ± 0.4
Follow up 4		49.6 ± 1.8
Follow up 5		80.4 ± 2.4
Follow up 6		130.6 ± 3.2

*GA* gestational age, *SD* standard deviation.

### Identification of patterns using the centering approach in GA-corrected growth data

**Figure 1:**
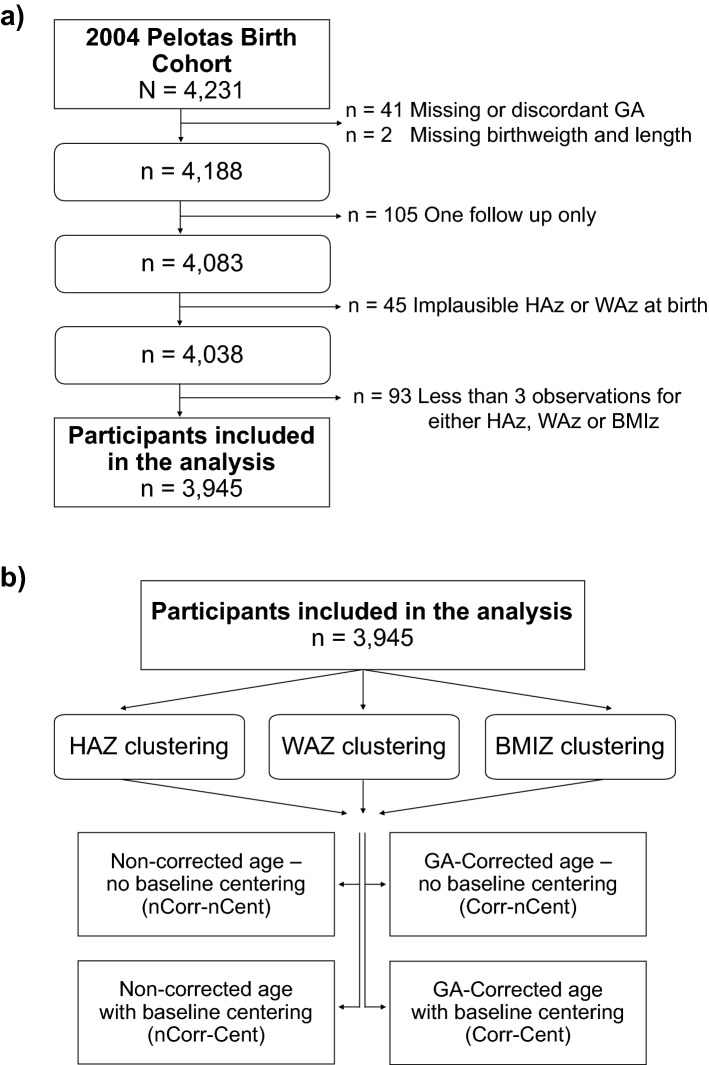
Flowchart of participants and analytical approaches tested. Participants were excluded if missing or discordant GA (n = 41), if missing or implausible birthweight or length variables (n = 47), if having only one follow up (n = 105), and those with < 3 observations for either HAz, WAz or BMIz (n = 93). GA, gestational age; HAZ, Height-for-age z-score; WAZ, Weight-for-age z-score; BMIZ, body mass index z-score.

Children with < 3 observations were excluded from the clustering analysis (n = 93), resulting in the inclusion of a minimum of four out of the seven time points available to trace their growth trajectories. This allowed us to retain 3945 (97.7%) of the total participants (see study Flow chart, Fig. 1a).

**Figure 2:**
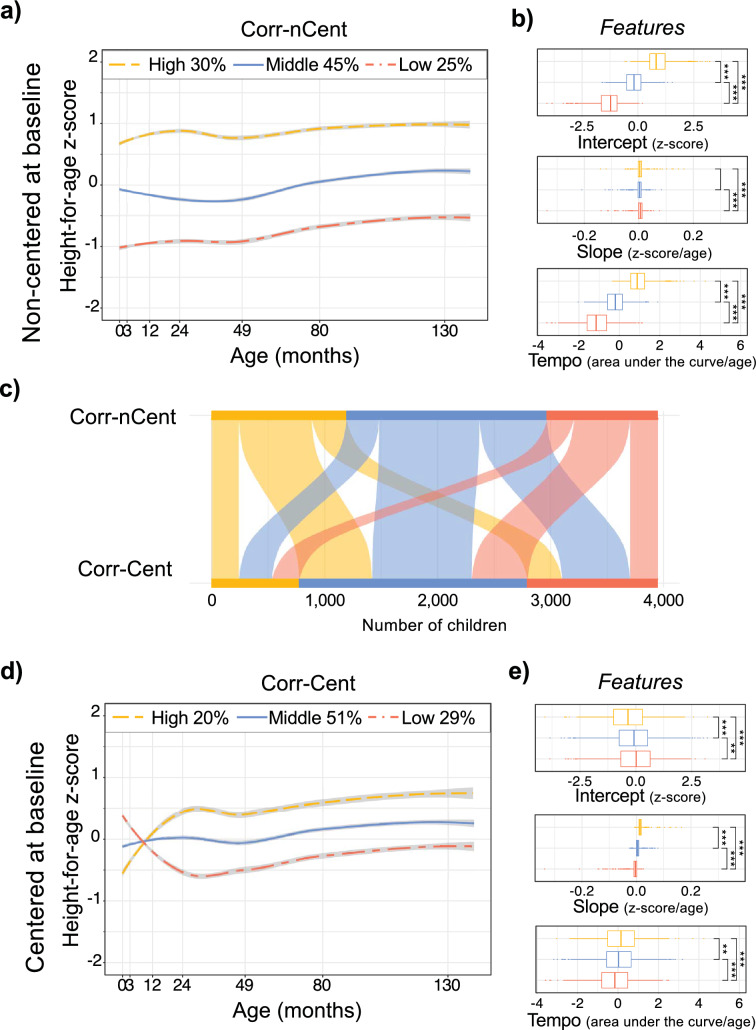
The impact of baseline centering on GA-corrected mean growth patterns of height-for-age z-score, 2004 Pelotas Birth cohort. (**a**) Growth patterns and (**b**) their features, identified with GA-correction and without baseline centering; (**d**) Growth patterns and (**e**) their features, identified with GA-correction and baseline centering. Percentage of children included in each pattern are indicated on top of each trajectory graph. (**b**,**e**) Features extracted from the linear model represent the starting point of the trajectory (intercept); the overall growth rate from birth to 11y (slope) and the average growth of the child per month (tempo). (**c**) Alluvial plot shows group mobility between mean *High*, *Middle* and *Low* growth patterns of height-for-age z-score between the analytical approaches, where the flow lines and numbers indicate children who change group (Supplementary Table S2). Mean differences in features assessed by non-parametric Kruskal–Wallis test and Dunn’s group comparison was used to assess mean differences. (p < 0.05). Corr-nCent, GA-corrected and non-centered at baseline; Corr-Cent, GA-corrected and centered at baseline; GA, gestational age.

Quality indices indicated the best number of clusters to be 2, however, we chose the second-best number of clusters (n = 3) for analyses, since this enhanced the difference between the two more distinct clusters (Supplementary Fig. S3). Three clusters provided an additional intermediate cluster. Three growth patterns were identified in the four analytical approaches for the three anthropometric z-scores (HAZ, Fig. 2 and Supplementary Fig. S4; WAZ and BMIZ, Supplementary Fig. S5). Children were grouped in “*High*”, “*Middle*” or “*Low*” patterns with an approximate split of a quarter, a half, and a quarter in each pattern, respectively (Supplementary Table S1). The mean z-score of the three growth patterns differed significantly across timepoints, regardless of the approach used with an average overall difference between groups of 0.34 SD in HAZ, 0.47 in WAZ, and 0.52 in BMIZ (data not shown). We did not find differences by sex among growth patterns, except in the corrected and centered approach for HAZ, where the *Low* pattern had a higher proportion of boys (16% vs 13%, data not shown). This discrepancy could be driven by the difference in pubertal timing earlier observed in girls. We performed a sensitivity analysis including children with less than 3 time points using the adjusted Rand index^10,11^, which resulted in clusters with moderate to an excellent agreement (range 0.72–0.92, data not shown)^12^.

Growth trajectories were summarized by their features, including intercept, slope, and tempo (Supplementary Figs. S6–8). The *High* trajectory pattern demonstrated the highest intercept (HAZ 0.64 ± 0.55 and 0.59 ± 0.63) and showed the highest tempo (HAZ 0.85 ± 0.53 and 0.82 ± 0.59). The *Middle* pattern was identified as having the lowest slope (HAZ 0.005 ± 0.012 and 0.005 ± 0.13). Meanwhile, the *Low* pattern showed the lowest intercept (HAZ − 1.85 ± 0.70 and 1.49 ± 0.75), and tempo (HAZ − 1.43 ± 0.70 and − 1.20 ± 0.67). Features for WAZ and BMIZ can be found in Supplementary Figs. S8 and 9.

### Differences in features of growth and group mobility detected with and without GA correction

GA-correction without baseline centering of HAZ did not influence the relatively flat horizontal mean growth patterns seen after 24 months of age (see Supplementary Fig. S4 vs. Fig. 2a). However, GA-correction increased the intercepts of the z-scores of all 3 patterns by a mean of 0.37 SD. This result was linked to moderate differences in the initial slope of the *High* and *Low* growth trajectories seen before 24 months of age, where GA-correction identified trajectories with initial slopes closer to zero. While most participants remained in the same trajectory patterns, 24% showed group mobility between *High* and *Middle* and between *Middle* and *Low* trajectories. Most children who changed pattern (n = 428, 10% of total sample) moved from the *Middle* pattern without GA-correction towards the *Low* growth pattern with GA-correction (Supplementary Table S2). For the group of preterm-born infants, 44.7% changed clusters with 19.1% reclassifying from *Middle* to *High* pattern, and 24.1% showing mobility between *Low* and *Middle* patterns. Percentages of reclassification across trajectories using the different approaches are presented in Supplementary Table S2.

### Differences in features of growth and group mobility detected with and without baseline centering

Compared to HAZ patterns obtained without centering, baseline centering resulted in more distinct overall mean trajectories, characterized by heterogeneous growth patterns in the first 2 years of life (see Supplementary Fig. S4). The children in the *High* pattern of non-GA-corrected centering approach started from the lowest intercept (HAZ − 0.75 ± 1.18) but showed the steepest positive slope (HAZ 0.02 ± 0.01) until reaching and maintaining the highest HAZ across the rest of childhood. While the mean *Middle* trajectory was similar with or without baseline centering, the mean *Low* trajectory captured with centering started from the highest intercept (HAZ − 0.33 ± 0.93) but decreased its z-score with a negative slope (HAZ − 0.003 ± 0.01) until HAZ stabilized at − 0.55 SD at 24 months of age to then gradually increase to − 0.27 SD by 11 years (Fig. 2d, and Supplementary Fig. S4c).

Importantly, group mobility was significant between *High*, *Middle* and *Low* patterns obtained with or without baseline centering, with most participants (64.2 and 65.3%) being reclassified (Supplementary Fig. S4 and Supplementary Table S2). This means that the centering approach provided a different set of children in each pattern. Group mobility was highest in both the *High* and *Middle* patterns. Interestingly, the type of group mobility between non-corrected age and centering non-corrected age was similar to that observed between the non-corrected and GA-corrected age, grouping more children in the *Low* pattern, but moving pre-term children from the *Low* to *Middle* and *High* patterns, though the percentage of mobility in the centering approach was greater. GA-correction of baseline centered HAZ showed a similar influence on mean patterns as seen with GA-correction without centering (i.e., differences in intercepts associated with slight changes in initial slope before 2 years of age). While baseline centering did reveal heterogeneity in growth rate before 2 years of age, the slopes of HAZ across all patterns (*High*, *Middle*, and *Low*) and analytical approaches were similar after 49 months for all conditions.

### Influence of baseline centering on growth trajectory group attribution of stunted children

**Figure 3:**
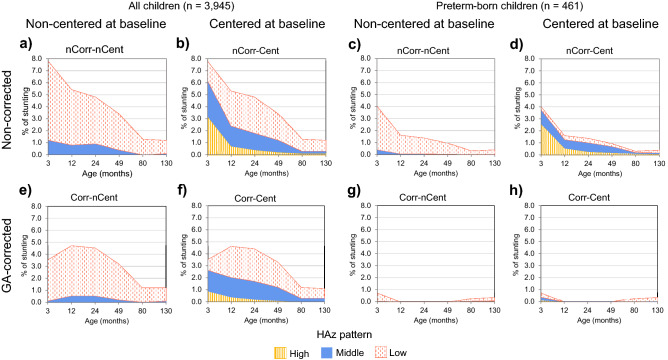
Percentage of children classified as stunted in each follow-up for all children and preterm-born in all approaches GA-corrected centered and non-centered clustering approaches. (**a**,**c**) Non-corrected, non-centered; (**b**,**d**) Non-corrected, centered; (**e**,**g**) GA-corrected, non-centered; (**f**,**h**) GA-corrected, centered. HAZ, height-for-age z-score; GA, gestational age.

We next evaluated the impact of baseline centering on estimations of stunting in groups with distinct growth patterns. We examined the group attribution of children classified as stunted (HAZ < − 2) at 3 months and 1, 2, 4, 6 and 11 years across the groups of *High*, *Middle* and *Low* growth patterns obtained from each of the 4 analytical approaches (Fig. 3).

With no baseline centering, most stunted children at any time point were grouped in the *Low* pattern, with only a small proportion (< 0.5% at all time points) classified in the *Middle* pattern (Fig. 3a). However, with baseline centering, stunted children were distributed among all three patterns (*Low*, *Middle* and *High*), regardless of age correction (Fig. 3). Thus, clusters identified using baseline centering helped identify groups that characterized stunting by early catch-up growth rates, showing either early improvements (*High* pattern), stable growth (*Middle* pattern), or experiencing growth declines (*Low* pattern) in early life. Interestingly, 70% (n = 91) of the children classified as stunted at 3 months in the *High* pattern with non-GA-corrected and centering approach were preterm-born (Fig. 3), reflecting the misclassification due to the use of the WHO-GS at birth for preterm-born children. However, this effect was not observed in the GA-corrected age approaches (Fig. 3e–h). Similar results were observed for underweight (WAZ < − 2), wasting (BMIZ < − 2), underweight (BMIZ > 2 and < 3) and obesity (BMIZ > 3) classifications (Fig. 3).

## Discussion

To the best of our knowledge, this is the first study that explores the impact of intercept and gestational age correction in the identification of children’s growth trajectories using a shape-based approach. We developed a pipeline and compared 4 analytical approaches which identified three distinct growth patterns (*High*, *Medium*, and *Low*), for 3 anthropometric z-scores (HAZ, WAZ, and BMIZ). While our analysis was conducted over a broad age range from birth to 11 years (i.e., pre/early adolescence), we found that the use of baseline centering highlighted sub-groups of children with distinct early growth patterns. GA-correction increased the intercept and reduced the slope of the trajectories between birth and 24 months of life for preterm children but had little impact on the overall classification of growth patterns. The baseline centering approach identified mean trajectories with early steeper slopes and classified stunted children that differed based on early catch-up growth rates. Thus, highlighting more contrasting early life features of growth trajectories (ie. long-term growth and direction of change), which would otherwise remain hidden as they are highly influenced by size at birth. This approach provided different grouping of children and could reveal child groups at risk of stunting in later childhood.

It has been previously shown that adjusting for differences in gestational age in the application of growth standards impacts the association between early growth and later outcomes by reducing standard errors specifically in Low- and Middle-Income Countries^13^. GA-correction has been applied previously in the identification of growth patterns in groups of preterm children only, in which heterogeneous post-term growth patterns were identified^14,15^. However, we found no study which compared the use of this age correction in the identification of growth patterns including pre-and term-born children. Although our results show that GA-correction can influence the membership of later childhood growth trajectory patterns, the observed impact on the mean patterns identified was very subtle affecting the mean trajectory only in the first 24 months. A notable contribution to the pattern classification was observed from GA-correction, which reduced the number of children classified as “stunted” in the *Low* pattern. Moreover, the number of children who were classified as “stunted” in the *High* pattern was lower in the first 12 months when GA-correction was applied; overall, this resulted in improving trajectories, which is possibly due to the “catch up” growth phenomenon often observed in this population. We did not explore the effect of applying GA-correction to all children (including term-born children), which could affect the classification of children born above 42 weeks GA (n = 243, 6% of total sample). Further epidemiological studies focusing on child growth should take into account this correction when information is available, to consider growth differences between term- and preterm-born children and its potential impact in the identification of growth trajectory patterns. Using clustering approaches is useful in epidemiological settings, where the identification of at-risk groups could inform the implementation of policies for interventions.

Complementing standard analysis of populational growth with techniques focused on capturing the heterogeneity of growth patterns could help sub-stratify at-risk children who could benefit from early intervention. Here, we show that using baseline centering before grouping children within trajectory patterns offers a focused perspective on the variation of early growth rates. By removing the weight of the starting point of the trajectory (centering) we were able to focus on the shape of the trajectories and capture the variation in growth that happens in the first 24 months of life. A similar normalizing approach has been used to identify patterns of crime over time and changes in antibody levels between animals of different farms^16,17^. Researchers showed that when correcting by the mean value of the farm the scale of the antibody effect and the variability between farms were removed^17^. Similar to the change in growth we captured in the patterns in our study, clustering by shape not only provided information on the direction of change, but also other complex nonlinear patterns which may be influenced by changes in time. We found that the centering approach allows for a shape-based grouping of growth trajectories, which differ mostly in their early life slope and were highly affected by the first 24 months of life, capturing the heterogeneity of early growth. In combination with GA-correction, baseline centering allowed to identify distinctive patterns of stunting that differ in early catch-up growth rates that could be linked to a height-for-age trajectory that is either improving (*High* pattern), continuous (*Middle* pattern), or declining (*Low* pattern). The impact that centering showed here can help identify early life features of the growth trajectories that would otherwise remain hidden as they are highly influenced by size at birth.

Among the limitations of this study are the use of the last menstrual period and Dubowitz methods to derive GA, which is less precise compared to early pregnancy ultrasound assessment. Moreover, the clustering approach tested here was not compared to other methods for clustering trajectories, although the performance of k-means using Fréchet distance is closely related to that of latent class growth analysis when trajectories vary smoothly with time^18^. However, the variability of clusters is expected within each clustering method and no standard for clustering has been established. Furthermore, we did not assess the association of the patterns identified in relation to prenatal, socioeconomic, or dietary variables. Yet, we analyzed growth trajectory patterns using a cohort study with high follow-up rates (> 85% at all time points) with high-quality data collection. We also tested this pipeline using three routinely applied growth standards used to assess child growth, which resulted in similar outcomes.

We were able to capture the growth patterns from childhood to early adolescence and confirm that even when removing the influence of the baseline values in the trajectory by centering, growth patterns are still predominantly defined by the first two years of life. The results of this study provide novel insights into the trajectories of growth among children that could identify important associations with prenatal and socioeconomic characteristics. This study supports previous recommendations of using GA-correction when assessing growth in epidemiological studies. This is especially important in research concerning early childhood and when the objectives include the assessment of undernutrition in the first year of life, or in populations where preterm birth and stunting are high. Moreover, we show the advantages of using the centering approach in studies where the outcome of interest could be conditional to the baseline values and influenced by the different patterns of growth that happen in early versus late childhood (such as consistent undernutrition through childhood). Refined methods that capture more nuanced variations in growth at different ages can support the determination of critical periods for growth and development of interventions.

## Methods

### Study design and participants

The 2004 Pelotas birth cohort is a prospective study of 4231 children born in 2004 in the urban area of Pelotas in southern Brazil^19^. Briefly, mothers were recruited at the five maternity hospitals in the region, covering 98% of all deliveries, after providing written informed consent. After birth, children were followed up longitudinally at months 3 (3.0 ± 0.1), 12 (11.9 ± 0.2), 24 (23.9 ± 0.4), and 48 (49.5 ± 1.7), and at 6 (6.8 ± 0.3) and 11 (11.0 ± 0.3) years of age. At each follow-up, anthropometry, socioeconomic, behavioural, and demographic data were collected by trained research staff. The 2004 Pelotas Birth cohort study was approved by the Research Ethics Committee of the Faculty of Medicine at the Universidade Federal de Pelotas for all follow-ups and written informed consent was obtained from the parents. Approval for the analyses included in this work was granted by the Ethics Committee from the University of Toronto (REB #36176) and the Hospital for Sick Children, Toronto (REB #1000059180). All analyses were performed in accordance with relevant guidelines and regulations.

Participants were excluded if missing or discordant GA (n = 41), if missing or implausible birthweight or length variables (n = 47), if having only one follow up (n = 105), and those with < 3 observations for either HAz, WAz or BMIz (n = 93) (Fig. 1a).

### Gestational age and postnatal age scale

Gestational age was estimated based on either the date of last menstruation as reported on a mother’s prenatal card or self-reported during the perinatal interview (n = 3316, 84%); or if missing, using the Dubowitz score at birth (n = 629, 16%), as, in Villar et al.^9^. Non-corrected standard postnatal age at each follow-up was calculated from the date of birth and the date of each visit. For children born pre-term (< 37 weeks GA, n = 461 (11.7%)), age was corrected for GA (GA-corrected age) by subtracting the standard postnatal age by the difference between the estimated GA at birth in days and the expected duration of a full-term pregnancy (i.e., 280 days): *GA-corrected age* = *[Postnatal age − (280 days − GA at birth)]*^20^.

### Anthropometric measurements

Details of anthropometric measurements were previously described^19,21,22^. Briefly, children’s length/height and weight were measured using standardized protocols^23^ by trained personnel. Length/height at birth, 3, 12, 24 and 48 months was measured using a foldable wooden anthropometer (with 1 mm precision). Height at 6 and 11 years was taken with a stadiometer (Harpenden) (maximum 2.06 m and 1 mm precision). Birthweight was measured using electronic pediatric scales with 10 g precision, and subsequent measurements were taken using an electronic scale (150 kg capacity and 100 g precision). At 3, 12 and 24-month visits, the mother’s and child’s weights were measured together, and the child’s final weight was calculated by subtracting the mother’s weight and the estimated weight of any remaining clothes. At the 4, 6 and 11-year visits, the child was weighted without shoes and wearing light clothes.

### Application of growth standards

Age- and sex-corrected z-scores for weight (WAZ) and length/height (HAZ) were calculated at birth and 3 months using the INTERGROWTH-21st growth standards^8^. WAZ, HAZ, and Body Mass Index z-scores (BMIZ) were calculated at each additional timepoint using WHO 2006 and 2007 references for children under or over 5 years of age, respectively^5,6^. z-score values were flagged for inspection if: 1) the absolute difference between timepoints was greater than 4 SD (n = 58 for non-corrected age z-scores; n = 54 for GA-corrected z-scores), or 2) WAZ < − 6 or > 5, HAZ < -6 or > 6, BMIZ < − 5 or > 5, as per WHO growth standards criteria (n = 138 for non-corrected age z-scores; n = 107 for GA-corrected z-scores). These flagged observations were set to missing when found to be outliers within the context of previous or subsequent timepoints (n = 76 observations for non-corrected age z-scores; n = 55 observations for GA-corrected z-scores) (see study Flow chart, Fig. 1a).

### Shape-based trajectory analysis

Shape-based trajectory analysis was conducted individually on each of the 3 anthropometric z-score measures using either non-corrected or GA-corrected age and with or without baseline centering.

#### Baseline centering

Baseline centering adjusts for the z-score value at birth by centering the intercept (i.e., initial value) to ~ 0 for all participants and was applied by subtracting 1.002 * the birth z-score value (HAZ/WAZ/BMIZ) to all the values in the trajectory: z-score – (1.002 * z-score at birth). For example, a child with a baseline z-score of ~ − 2 at birth would have a baseline-centred z-score of ~ 0; the values of the trajectory would thus be displaced in the z-score scale but would maintain the same shape (Supplementary Fig. S1). The baseline values were transformed to ~ 0 since an exact zero value could not be used to compute the distance matrix.

#### Clustering pre-configuration

The number of relevant clusters was chosen based on the assessment of 24 different quality indexes (Supplementary Fig. S3) obtained by varying all combinations of number of clusters, using the NbClust R package^24^ and manual inspection.

#### Clustering of trajectories

Clustering analysis was conducted using k-means with the kmlShape R package^12^ and using the shape-respecting generalized Fréchet’s distance metric, a similarity measure for geometric shapes. This method is defined on a continuous interval and thus allows for missing data and unequal interval sampling^12,25^. Each cluster is referred to as a growth pattern. The clustering approach was set to iterate until clusters become stable. For the visual representation of results, the mean trajectory for each identified pattern was traced with the least-squares non-parametric locally weighted smoothing (LOWESS) function.

We conducted 4 parallel growth clustering analyses (Fig. 1b): (1) non-corrected age (without GA-correction)—without baseline centering; (2) non-corrected age (without GA-correction)—with baseline centering; (3) GA-corrected age—without baseline centering; and (4) GA-corrected age—with baseline centering. The combinations of these approaches created 12 sets of clustering results (4 for each anthropometric indicator). For the centering approach, we kept the pattern membership but used the original (un-transformed) z-scores to model the mean trajectories, allowing us to compare the results with the non-centering approach (Supplementary Fig. S2). We used alluvial plots to visualize the mobility of children between the patterns identified with each analytical approach. All analyses were implemented in R (version 3.6.1).

### Characterization of growth features and attribution of malnutrition between patterns

To characterize the different growth clusters, we extracted linear model features for each child’s trajectory for HAZ, WAZ and BMIZ:

- intercept: starting point of the trajectory/initial measure,
- slope: slope of the linear trajectory, the overall growth rate from birth to 11y, and;
- tempo: the average growth of the child per month, calculated as the total area under the curve of the linear model divided by the child’s age in months.

For each growth measure, the non-parametric Kruskal–Wallis test followed by Dunn’s for group comparison was used to assess mean differences in features between children with distinct growth patterns.

To explore the application of the analytical approaches in the identification of groups of children at risk of malnutrition through childhood, we used a chi-squared test to compare the proportion of children classified as stunted (HAZ < − 2), underweight (WAZ < − 2), wasted (BMIZ < − 2), overweight (BMIZ > 2) and with obesity (BMIZ > 3) in each pattern among clustering approaches.

## Supplementary Information

Supplementary Information.

## Data Availability

The data that support the findings of this study are available from the 2004 Pelotas Birth cohort study investigators, but restrictions apply to the availability of these data, which were used under license for the current study, and so are not publicly available. Data are however available upon reasonable request and with permission of the 2004 Pelotas Birth cohort research committee. Example analytic code is posted on GitHub at the following URL: https://github.com/Comelli-lab/shape-based-approach-to-identify-gestational-age-adjusted-growth-patterns.
