# Supplementary material for: A novel shape-based approach to identify gestational age-adjusted growth patterns from birth to 11 years of age
Source: Sci Rep. 2023 Jan 31;13:1709. doi: 10.1038/s41598-023-28485-4 (PMC9889302; doi:10.1038/s41598-023-28485-4)

Title: A novel shape-based approach to identify gestational age-adjusted growth patterns from birth to 11 years of age

López-Domínguez, Lorena [a, b]; Bassani, Diego G [c, d, e]; Bourdon, Celine [b, f]; Massara, Paraskevi [a, b]; Santos, Iná S [g], Matijasevich, Alicia [g, h]; Barros, Aluísio JD [g]; Comelli Elena M [a, i, *], Bandsma, Robert HJ [a, b, d, j, *].

[a] Department of Nutritional Sciences, Faculty of Medicine, University of Toronto, Toronto, ON, Canada.

[b] Translational Medicine Program, Hospital for Sick Children, Toronto, ON, Canada

[c] Department of Epidemiology, Dalla Lana School of Public Health, University of Toronto, Toronto, ON, Canada.

[d] Centre for Global Child Health, Child Health Evaluative Sciences, Hospital for Sick Children, Toronto, ON, Canada.

[e] Division of Paediatric Medicine, Hospital for Sick Children, Toronto, ON, Canada.

[f] The Childhood Acute Illness & Nutrition Network, Nairobi, Kenya.

[g] Post-Graduate Program in Epidemiology, Federal University of Pelotas, Pelotas, RS, Brazil.

[h] Department of Preventive Medicine, Faculty of Medicine, University of São Paulo, SP, Brazil.

[i] Joannah and Brian Lawson Center for Child Nutrition, University of Toronto, Toronto, ON, Canada.

[j] Division of Gastroenterology, Hepatology and Nutrition, Hospital for Sick Children, Toronto, ON, Canada.

*co-corresponding authors: Robert Bandsma robert.bandsma@sickkids.ca, Elena Comelli elena.comelli@utoronto.ca

Baseline centering

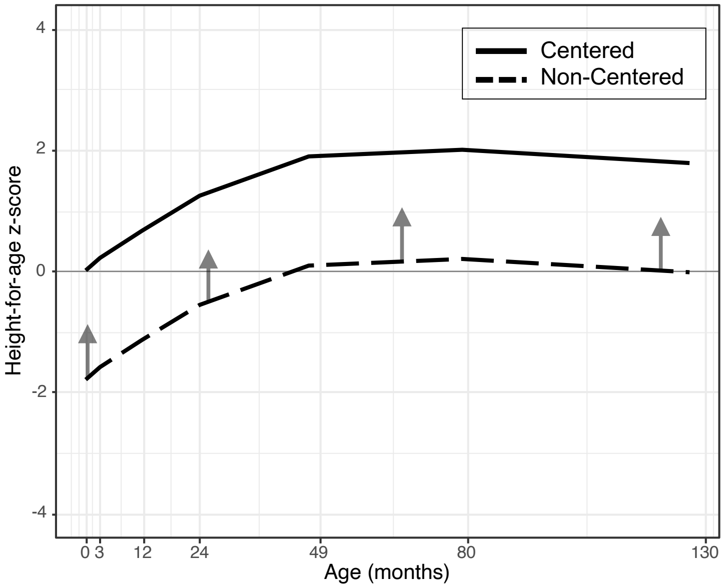

Supplementary Figure S1 Example of the effect of centering on an individual growth trajectory. The dashed line illustrates the original non-centered trajectory. The arrows represent the direction of the trajectory displacement after centering at baseline

With this transformation, we clustered the trajectories using k-means and Fréchet distance. After patterns were identified we modeled the mean trajectory for each cluster (Supplementary Figure S2A). To compare the obtained patterns with those from the non-centered approach we modeled the trajectories keeping the pattern membership but using the original (un-transformed) Z-scores (Supplementary Figure S2B). This positioned the means in a different place on the Z-score scale but maintaining the same shape.

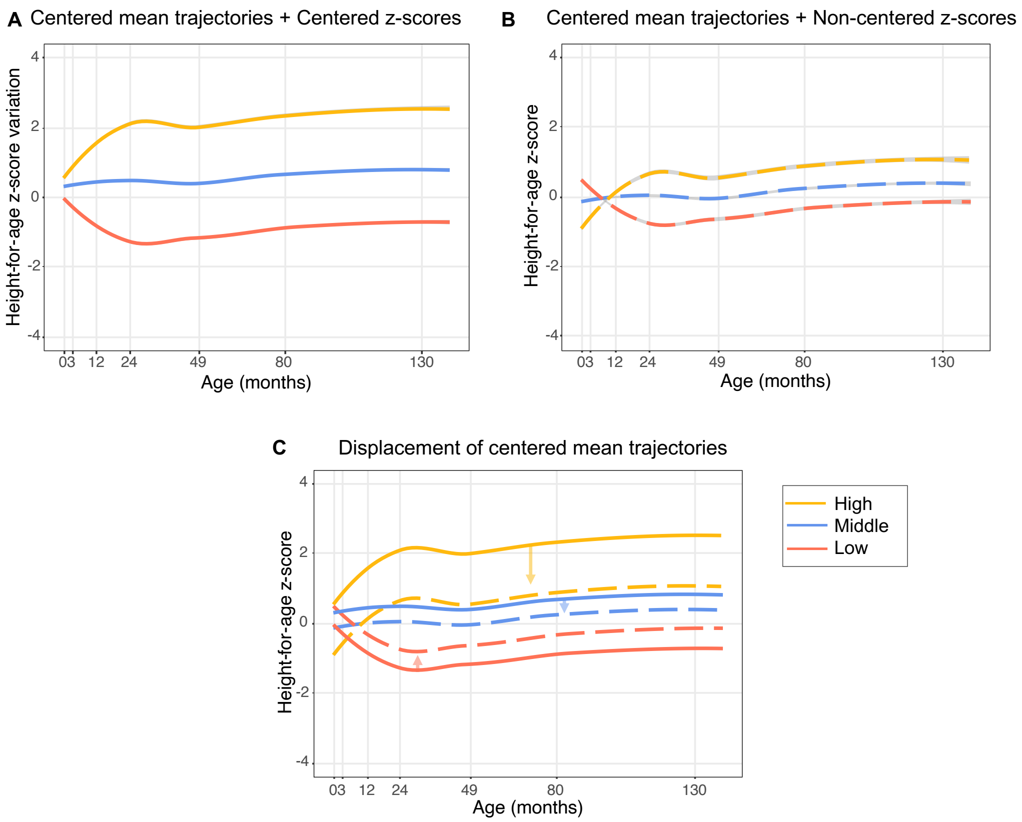

Supplementary Figure S2 Mean trajectory patterns obtained using GA-corrected age and centered Z-scores. A) Centered mean trajectories with centered Z-scores. B) Centered mean trajectories after plotted with non-centered Z-scores C) Displacement of centered mean trajectories after plotting using non-centered Z-scores. Solid lines represent the position of the cluster's mean trajectories when using centered Z-scores. Dashed lines represent the position of the cluster's mean trajectories when using original Z-scores

Supplementary Figure S3 Indices assessed for optimal number of clusters suggested for height-for-age z-scores using NbClust R package

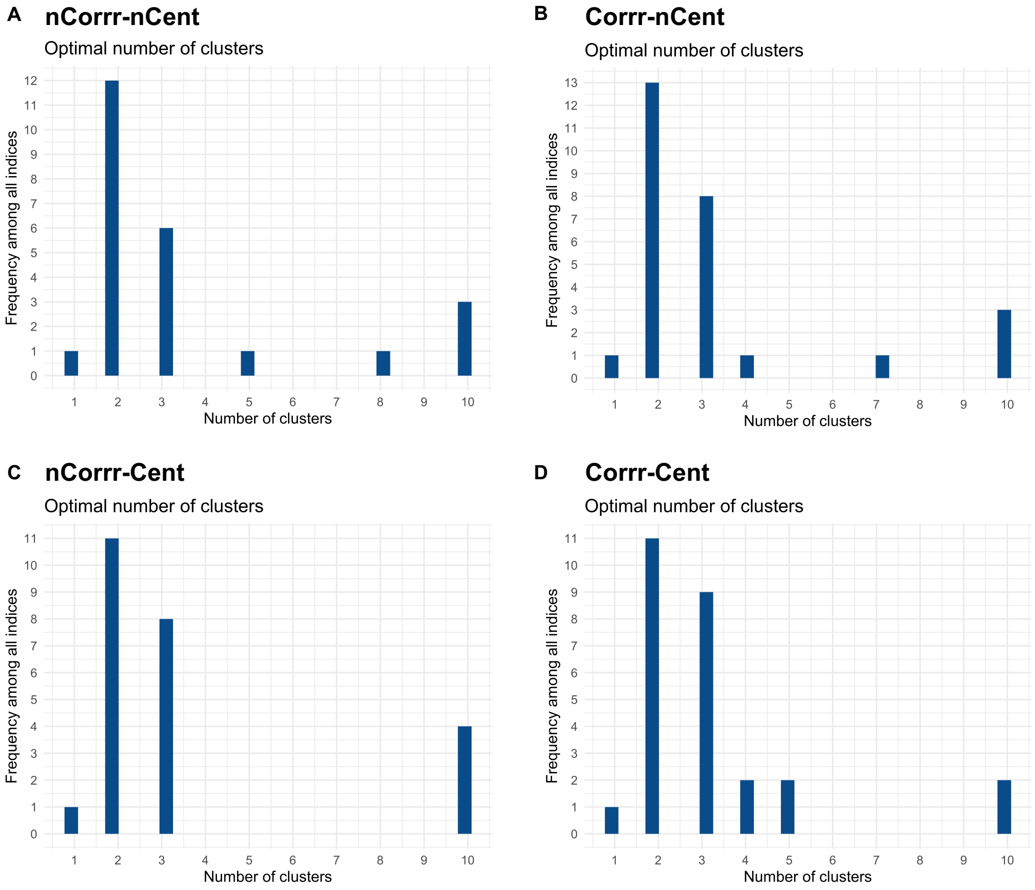

Indices used: Krzanowski and Lai index, Calinski and Harabasz index, Hartigan index, Cubic Clustering Criterion, Scott index, Marriot index, TraceCovW index, TraceW index, Friedman index, Rubin index, C-index, Davies and Bouldin index, Silhouette index, Duda index, Pseudot2 index, Beale index, Ratkowsky index, Ball index, Gap index, Frey index, Mcclain index, Dunn index, SDindex, SDbw index. We chose the second-best number of clusters (3) for all analytical approaches.

Supplementary Table S1. Number of children (percentage) classified in each HAZ, WAZ, and BMIZ pattern, among different conditions.

	Non-centered at baseline		Centered at baseline	
Growth Patterns	Non-Corrected age (nCorr-nCent)	Corrected age (Corr-nCent)	Non-corrected age (nCorr-Cent)	Corrected age (Corr-Cent)
HAZ				
- High	1225 (31.1%)[a]	1194 (30.3%)[a]	715 (18.1%)[b]	775 (19.6%)[b]
- Middle	2035 (51.6%)[a]	1773 (44.9%)[b]	1984 (50.3%)[a]	2018 (51.2%)[a]
- Low	685 (17.4%)[a]	978 (24.8%)[b]	1246 (31.6%)[c]	1152 (29.2%)[c]
WAZ				
- High	992 (25.1%)[a]	825 (20.9%)[b]	510 (12.9%)[c]	565 (14.3%)[c]
- Middle	2019 (51.2%)[a]	1953 (49.5%)[a]	2046 (51.9%)[a]	1937 (49.1%)[a]
- Low	934 (23.7%)[a]	1167 (29.6%)[b]	1389 (35.2%)[c]	1443 (36.6%)[c]
BMIZ				
- High	778 (19.7%)[a]	996 (25.2%)[b]	933 (23.7%)[b]	894 (22.7%)[b]
- Middle	1869 (47.4%)[a]	1720 (43.6%)[b]	2338 (59.3%)[c]	1862 (47.2%)[a]
- Low	1298 (32.9%)[a]	1229 (31.2%)[a]	674 (17.1%)[b]	1189 (30.1%)[a]

nCorr-nCent, Trajectories calculated using non-corrected age; Corr-nCent, Trajectories calculated using corrected age; nCorr-Cent, Trajectories calculated using non-corrected age and centered on baseline measurement; Corr-Cent, Trajectories calculated using corrected age and centered on baseline measurement. Chi-square test: different letters indicate differences between approaches ($p<0.05$).

Supplementary Figure S4 The effect of baseline centering on mean growth patterns of height-for-age z-score (not GA-corrected). A) without GA-correction or baseline centering (nCorr-nCent); C) without GA-correction with baseline centering (nCorr-Cent). Percentage of children included in each pattern are indicated on top of each trajectory graph. B) Alluvial plot shows group mobility between mean *High*, *Middle* and *Low* growth patterns of height-for-age z-score as obtained by centering approach, where the flow lines indicate children who change group. *High* pattern is presented in yellow, *Middle* in blue and *Low* pattern in red

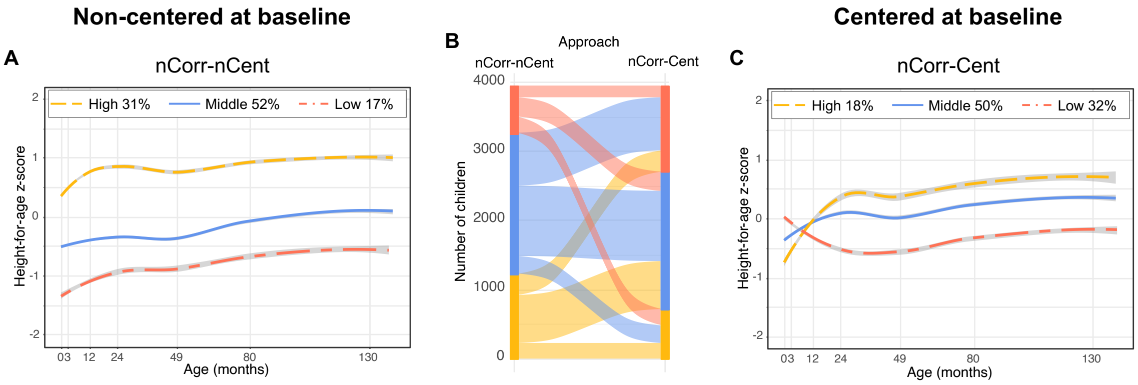

Supplementary Figure S5 Mean trajectory patterns of weight-for-age and BMI-for-age z-scores and percentage of sample included in each pattern, presented by age correction and centering approach. *High* pattern is represented in yellow; *Middle* in blue and *Low* in red. Abbreviations: WAZ, height-for-age z-score; BMIZ, BMI-for-age z-score

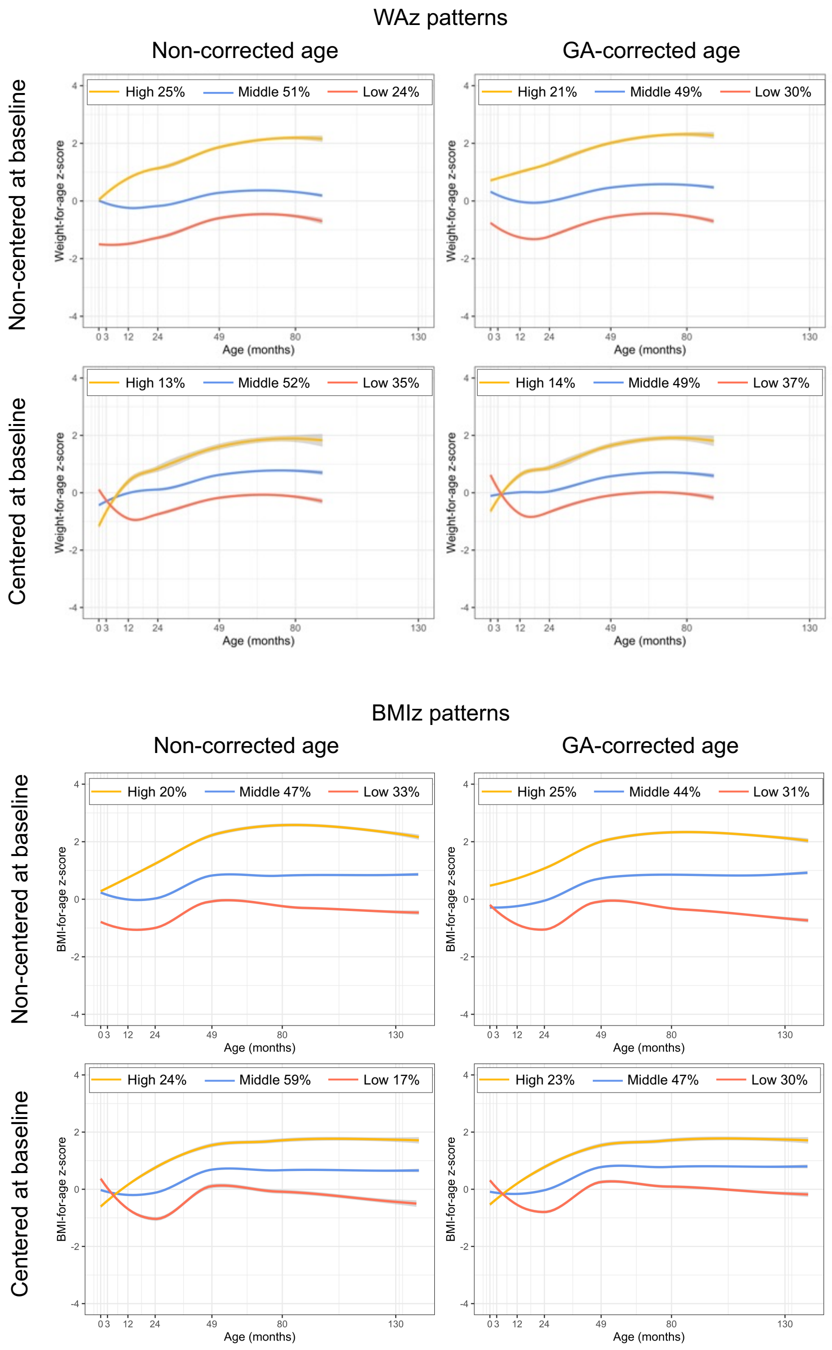

Supplementary Table S2. Participants changing HAZ growth pattern classification among age-correction and centering approaches.

**A.** Non-corrected age vs GA-corrected age (nCorr-nCent vs Corr-nCent)				
Mobility patterns	Total (n)	Total (% of pattern)	Preterm born (% of reclassified)	Preterm born (% of total preterm)
High → High	1014	82.8	4.3	9.5
High → Middle	211	17.2	0.0	0.0
High → Low	0	0.0	0.0	0.0
Middle → High	174	8.6	50.6	19.1
Middle → Middle	1433	70.4	7.1	22.1
Middle → Low	428	21.0	0.2	0.2
Low → High	6	0.9	100.0	1.3
Low → Middle	129	18.8	86.0	24.1
Low → Low	550	80.3	19.8	23.6

**B.** Non-corrected age centered vs GA-corrected age centered (nCorr-Cent vs Corr-Cent)				
Mobility patterns	Total (n)	Total (% of pattern)	Preterm born (% of reclassified)	Preterm born (% of total preterm)
High → High	503	70.3	17.9	19.5
High → Middle	191	26.7	55.0	22.8
High → Low	21	2.9	100.0	4.6
Middle → High	272	13.7	0.0	0.0
Middle → Middle	1462	73.7	4.4	13.9
Middle → Low	250	12.6	48.4	26.2

Low → High	0	0.0	0.0	0.0
Low → Middle	365	29.3	0.0	0.0
Low → Low	881	70.7	6.8	13.0

**C.** Non-corrected age vs Non-corrected age centered (nCorr-nCent vs nCorr-Cent)				
Mobility patterns	Total (n)	Total (% of pattern)	Preterm born (% of reclassified)	Preterm born (% of total preterm)
High → High	227	18.5	6.6	3.3
High → Middle	690	56.3	3.2	4.8
High → Low	308	25.1	2.3	1.5
Middle → High	254	12.5	25.6	14.1
Middle → Middle	1015	49.9	9.0	19.7
Middle → Low	766	37.6	4.6	7.6
Low → High	234	34.2	58.1	29.5
Low → Middle	279	40.7	25.8	15.6
Low → Low	172	25.1	10.5	3.9

**D.** GA-corrected age vs GA-corrected age centered (Corr-nCent vs Corr-Cent)				
Mobility patterns	Total (n)	Total (% of pattern)	Preterm born (% of reclassified)	Preterm born (% of total preterm)
High → High	242	20.3	8.7	4.6
High → Middle	643	53.9	10.1	14.1
High → Low	309	25.9	16.8	11.3
Middle → High	292	16.5	13.7	8.7

Middle → Middle	882	49.7	7.8	15.0
Middle → Low	599	33.8	17.4	22.6
Low → High	241	24.6	12.0	6.3
Low → Middle	493	50.4	7.1	7.6
Low → Low	244	24.9	18.9	10.0

HAZ, height-for-age Z-score; nCorr, no GA-corrected age; Corr, with GA-corrected age; nCent, no baseline centering; Cent, with baseline centering; HAz, height-for-age z-score; GA, gestational age. Arrows indicate mobility between clusters. Colors are used consistently with Figure 3.

Supplementary Figure S6 Comparison of features describing the HAZ growth trajectory patterns in the non-corrected approach among centering approaches

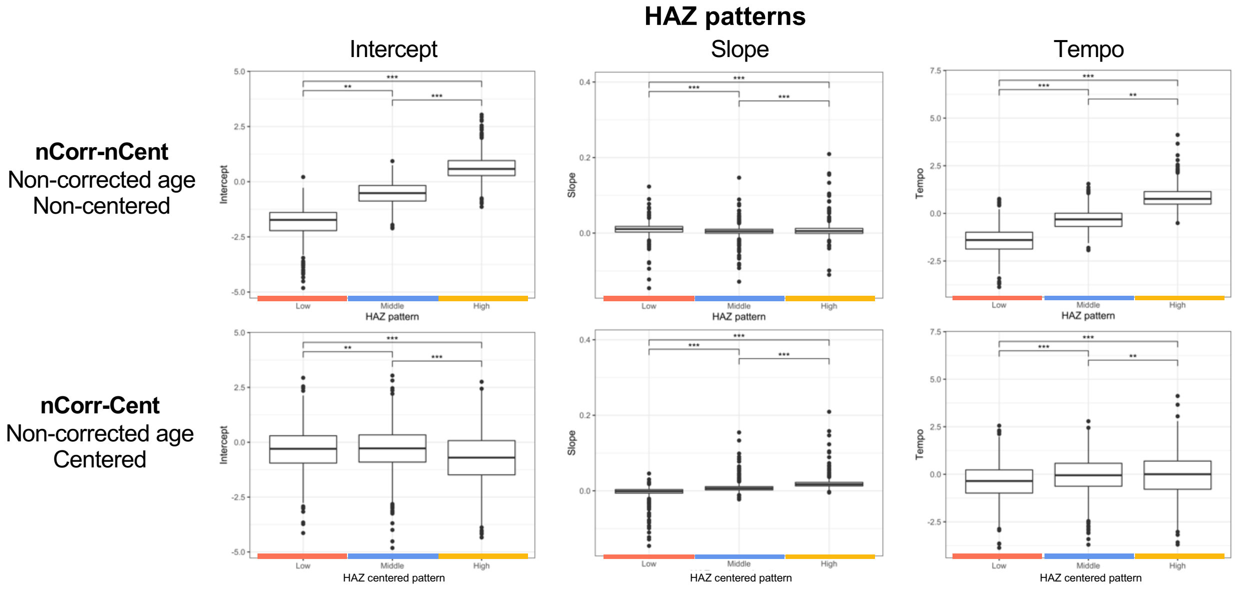

Supplementary Figure S7 Comparison of features describing the HAZ growth trajectory patterns in the GA-corrected approaches and divided from birth to 24 months and >24months.

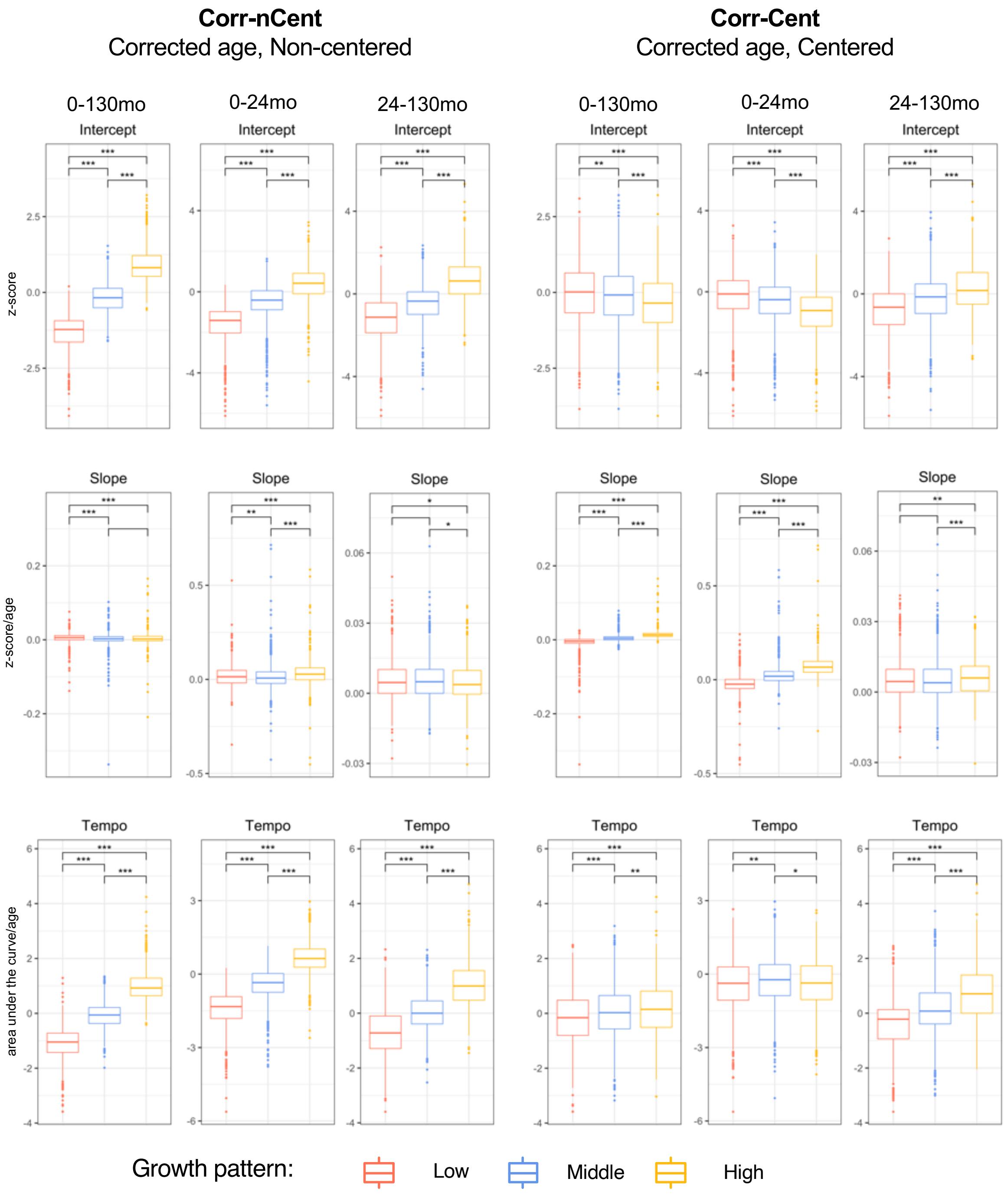

Features were extracted by modeling children's growth trajectories as linear models: intercept - starting point of the trajectory/initial measure (z-score value), slope - slope of the linear trajectory (z-score value/age in months), the overall growth rate from birth to 11y, and; tempo - the average growth of the child per month, calculated as the total area under the curve of the linear model divided by the child's age in months.

Supplementary Figure S8 Comparison of features describing the WAZ growth trajectory patterns between corrected age and centering approaches

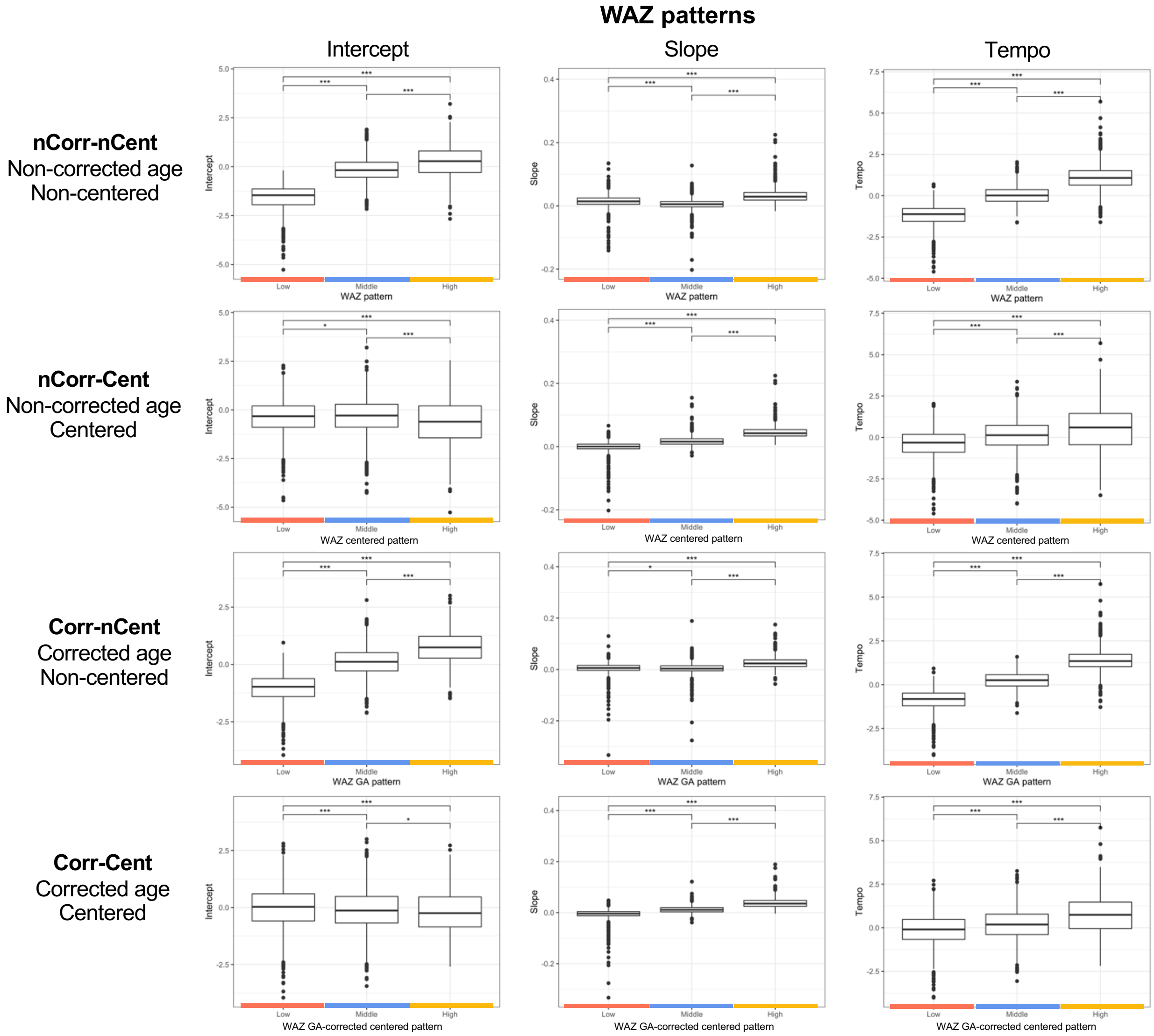

Supplementary Figure S9 Comparison of features describing the BMIZ growth trajectory patterns between corrected age and centering approaches

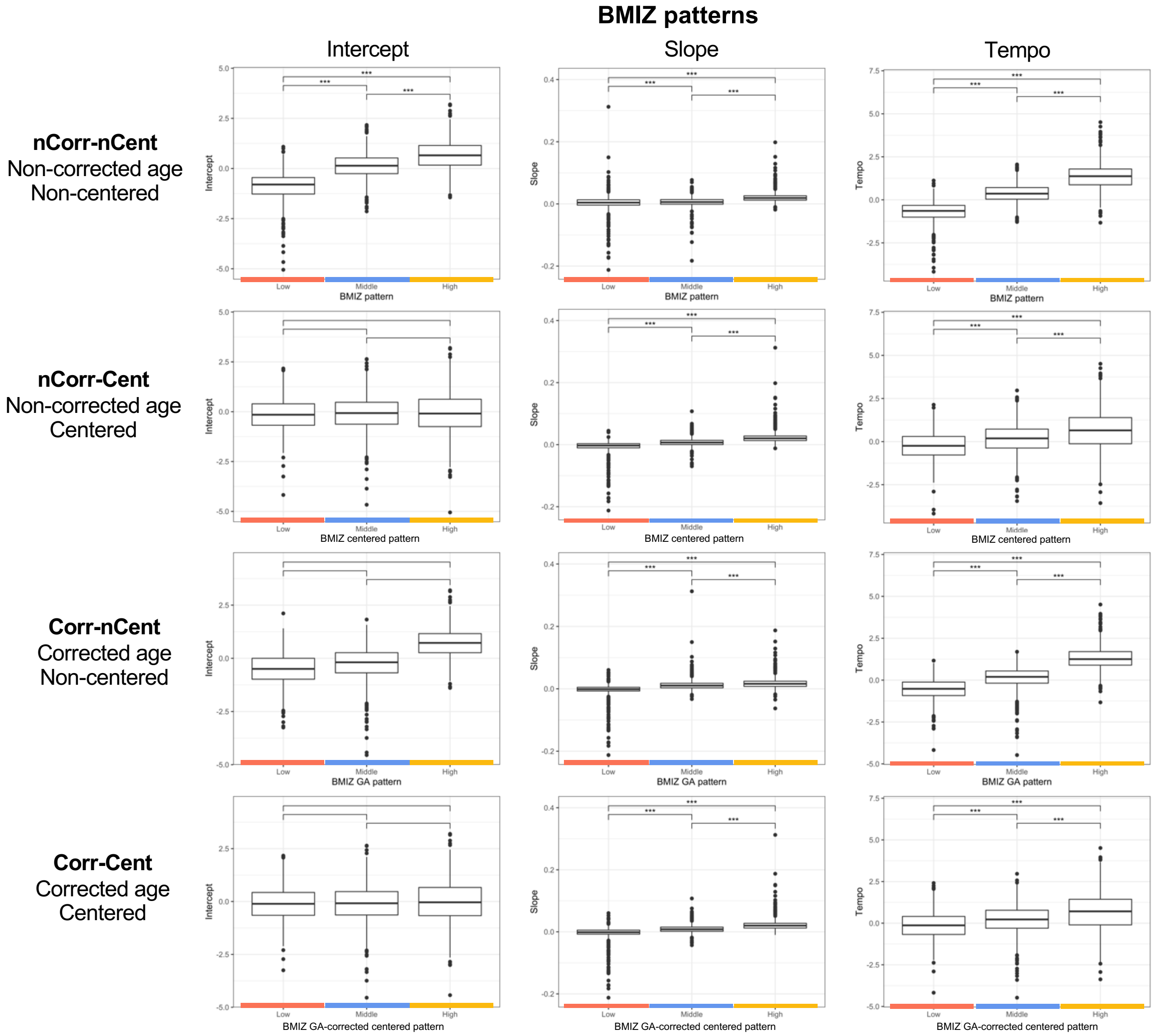

Supplementary Figure S10 Classification of children according to WAz and BMIz in each follow-up for each analytical approach. A) Percentage of children classified as underweight (WAZ < -2); B) Percentage of children classified as wasted (BMIZ < -2); C) Percentage of children classified as overweight (BMIZ > 2 & < 3); D) B) Percentage of children classified as obese (BMIZ > 3)

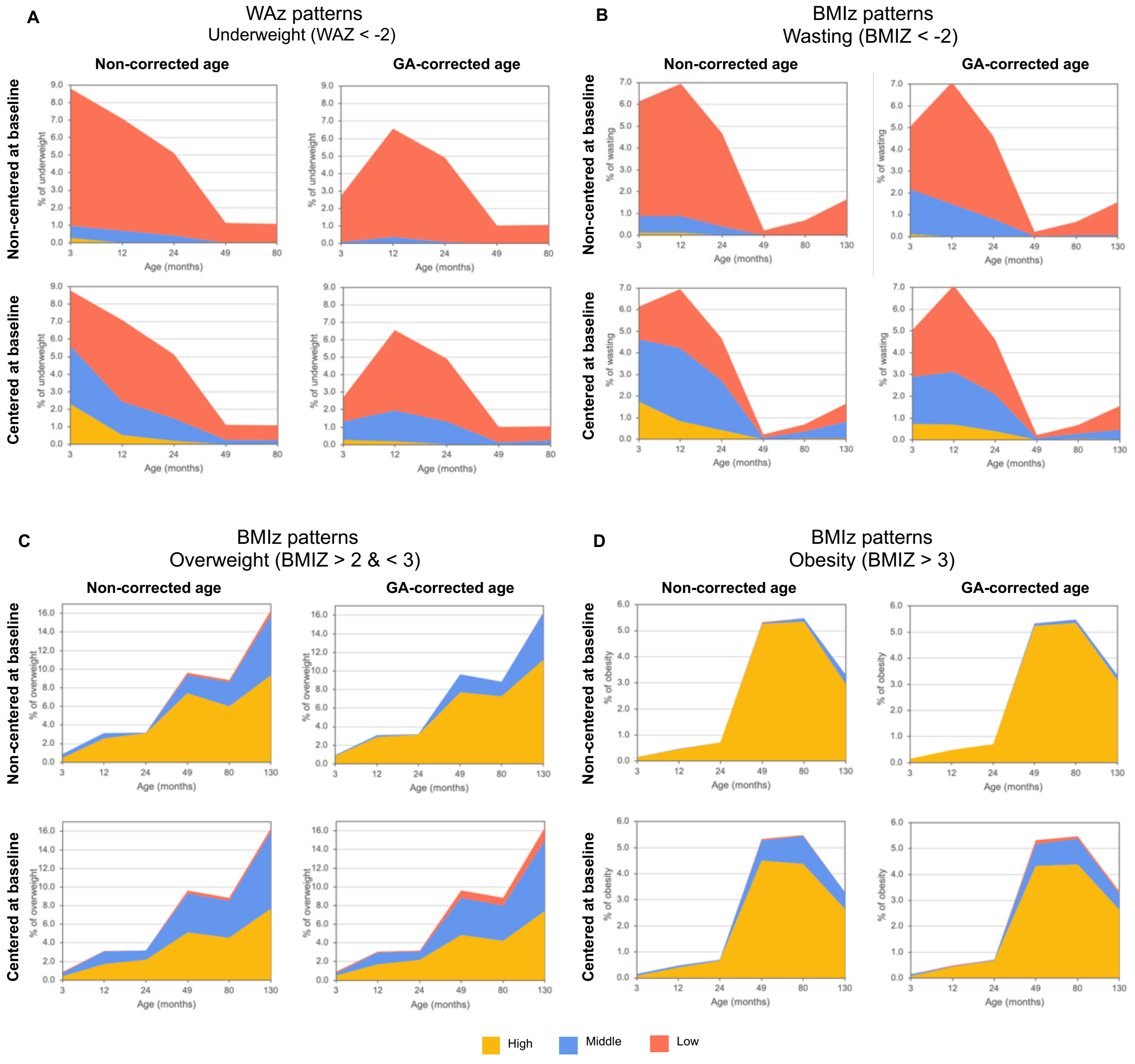

Supplement: Supplementary file 1 — Supplementary Information. [file 41598_2023_28485_MOESM1_ESM.pdf]
